# The Immune Microenvironment in Brain Metastases of Non-Small Cell Lung Cancer

**DOI:** 10.3389/fonc.2021.698844

**Published:** 2021-07-14

**Authors:** Lumeng Luo, Peiyi Liu, Kuaile Zhao, Weixin Zhao, Xiaofei Zhang

**Affiliations:** ^1^ Department of Radiation Oncology, Fudan University Shanghai Cancer Center, Shanghai, China; ^2^ Department of Oncology, Shanghai Medical College, Fudan University, Shanghai, China; ^3^ Department of Orthopedics, TongRen Hospital, School of Medicine Shanghai Jiao Tong University, Shanghai, China

**Keywords:** immune microenvironment, brain metastases, non-small cell lung cancer, immune therapeutics, brain metastases of non-small cell lung cancer

## Abstract

Brain metastasis of non-small cell lung cancer is associated with poor survival outcomes and poses rough clinical challenges. At the era of immunotherapy, it is urgent to perform a comprehensive study uncovering the specific immune microenvironment of brain metastases of NSCLC. The immune microenvironment of brain is distinctly different from microenvironments of extracranial lesions. In this review, we summarized the process of brain metastases across the barrier and revealed that brain is not completely immune-privileged. We comprehensively described the specific components of immune microenvironment for brain metastases such as central nervous system-derived antigen-presenting cells, microglia and astrocytes. Besides, the difference of immune microenvironment between brain metastases and primary foci of lung was particularly demonstrated.

## Background

Brain metastases are the most common type intracranial tumors, which are commonly metastasized from lung cancer (1). Approximately 50% of brain metastases originate from non-small cell lung cancer (NSCLC). During the progression of NSCLC, about one third of patients may develop brain metastases (1, 2). Current therapeutic strategies for brain metastases of NSCLC are largely limited, and the prognosis is relatively poor because of the specific anatomic and physiologic features of the central nervous system (CNS). Moreover, comprehensive researches on brain metastases of NSCLC are significantly lacked. Immunotherapy has been rapidly adopted for the treatment of NSCLC (3, 4). Recent small-scale clinical studies have shown that some of NSCLC patients can be benefited from immune checkpoint inhibitors (5). However, due to genetic differences between brain metastases and primary tumors, as well as the difference in tumor microenvironment, the response of intracranial and extracranial lesions to systemic immunotherapy may differ a lot. In addition, the difficulty in collecting intracranial tissues increases the challenge in clarifying the molecular mechanism of brain metastases (6). Therefore, it is urgent to carry out an in-depth exploration on the immune microenvironment of brain metastases, aiming to guide clinical treatment.

## The Process of Brain Metastases

**Figure 1:**
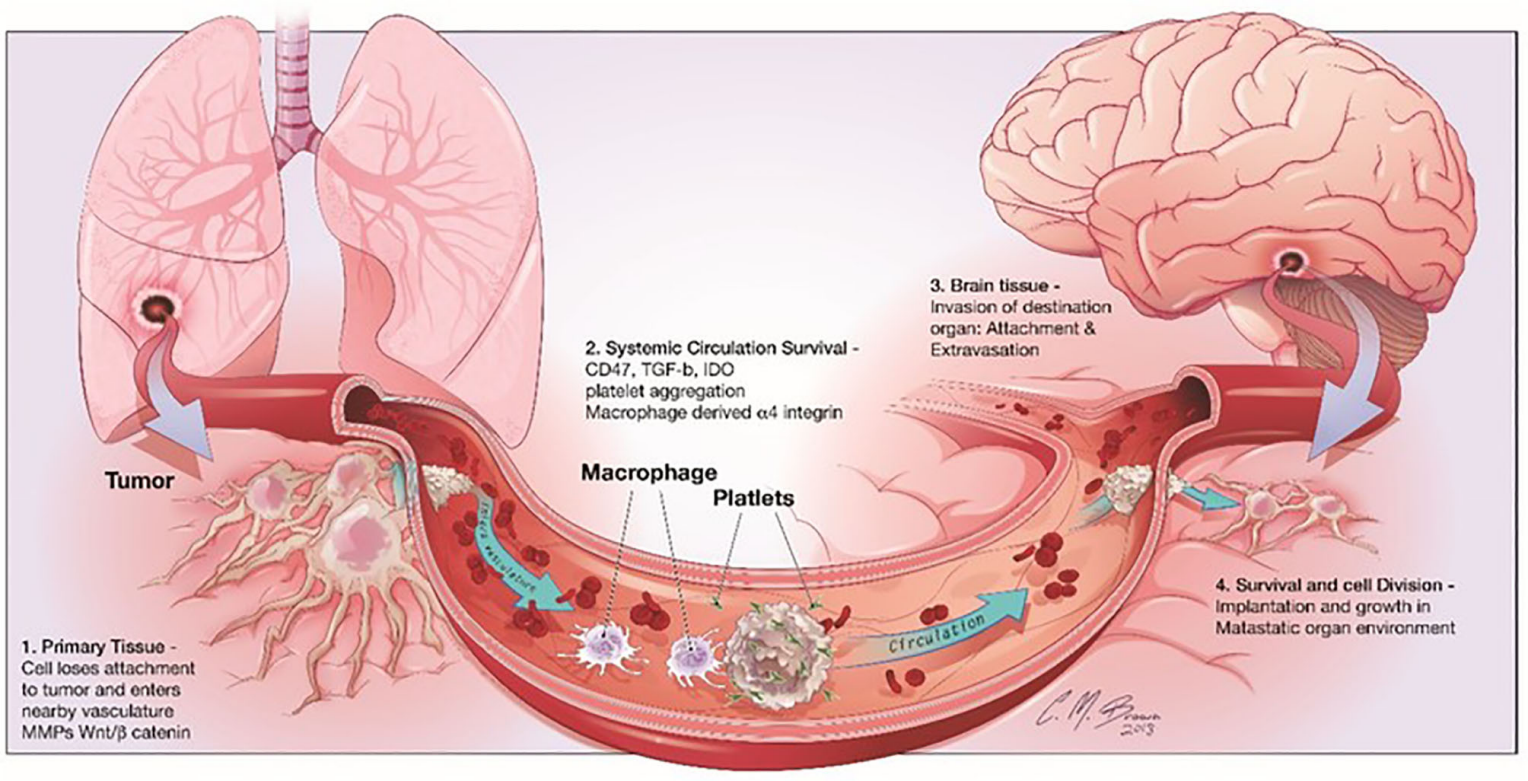
Steps involved in brain metastasis. Brain metastasis cascade involves four major steps: 1) Detachment of the metastatic cell from the primary cancer, 2) Survival in systemic circulation, 3) Invasion in the brain parenchyma and 4) Survival in the CNS microenvironment.

Cancer metastasis is one of the most significant characteristics of malignant tumors, which is a multistep cell-biological process, called the invasion-metastasis cascade (7). During metastatic progression, tumor cells detach from their primary lesions (locally invasive and intravasate), translocate systemically (survive in the circulation, arrest at a distant tissue and extravasate), and finally form the metastases in the foreign microenvironment of distant organs (**Figure 1**) (7). Although the circulating tumor cells (CTCs) in the hematogenous circulation could disseminate to a variety of secondary loci, it is noticed that the metastases of a certain type of carcinoma could only form in particular target organs (7). In 1889, Stephen Paget proposed the well-known “seed-and-soil” hypothesis of metastases that metastases only develop at those organ sites (“soils”), in which the newly “seeded” metastatic tumor cells are suitably growing (8). The nervous system is one of the most preferential and frequent metastatic sites of NSCLC (9). CTCs infiltrate through the blood circulation at brain capillaries with a slower flow rate, where they interact with microvascular endothelial cells and secrete cytokines. Then, tumor cells with a strong invasiveness ability circulate through the bloodstream, the brain lymphatics or the CSF, and survive and thrive in the parenchymal, leptomeningeal, or epidural areas, thus leading to brain metastases.

## The Molecular Mechanism of Brain Metastases of NSCLC

The molecular mechanisms underlying brain metastases of NSCLC remain largely unknown due to the lack of *in vitro* models that simulate the complex structure and microenvironment of CNS. A previous study established a multi-organ microfluidic bionic chip platform to recapitulate the process of brain metastases. It is found that AKR1B10 can promote the extravasation of lung cancer cells through the blood–brain barrier (BBB) and then induce brain metastases (10). Through comparing genomic sequencing data of a large number of brain metastases and primary lung adenocarcinomas, three novel metastatic drivers with significantly higher amplification frequencies are identified, including MYC, YAP1, and MMP13. Overexpression of them increases the incidence of brain metastases (11, 12). Besides, *in vitro* and *in vivo* experiments demonstrated that cell adhesion molecule 2 (CADMA2), long noncoding RNA MALAT1, and microRNA-330-3p promote the development of brain metastases by inducing epithelial-mesenchymal transition (EMT) in NSCLC (13–15). The transmembrane cell adhesion protein ADAM9 is able to promote lung cancer metastases to the brain by a plasminogen activator-based pathway (16). Moreover, activated leukocyte cell adhesion molecule (ALCAM), the tubulin-detyrosinating activity of VASH1, lysophosphatidylcholine acyltransferase 1 (LPCAT1), and the TAZ-AXL-ABL2 feed-forward signaling axis are also essential for the formation of brain metastases from NSCLC (17–20). As for immune-related mechanism, tumor-induced peripheral immunosuppression might promote brain metastases in patients with NSCLC (21). Patients with brain metastatic lung carcinoma exhibit a profound systemic immunosuppression with increased myeloid-derived suppressor cells, regulatory T cell populations, peripheral monocyte PD-L1, myeloid-derived suppressor cells (MDSCs), and regulatory T cells compared to early stage pre-metastatic patients and healthy controls, accompanied by less reactive T cells and worse survival (21).

## Incompletely Immune Privileged CNS

There are three main barriers in CNS, including the blood–brain barrier (BBB), blood–cerebrospinal fluid (BCSFB) barrier, and the blood– tumor barrier (BTB) (22). In the normal brain, BBB and BCSFB are the initial gatekeepers of CNS, which are formed by the tight junctions of the endothelial cells of capillaries and connective tissues and responsible for protecting CNS from a massive inflammation (brain edema) (23). CTCs could cross the barrier by the transendothelial migration. When the micrometastases (<1 mm) are formed, the BBB still functions normally to protect the micrometastases escaping from the effective anti-cancer drugs, such as water-soluble agents and macromolecules (23, 24). The formation of metastatic tumor in the brain leads to neo-angiogenesis, vascular remodeling, and changes of surface molecules, such as overexpression of the pericyte protein desmin and deficiency of physiological TJ protein (25–27). The changed neurovascular-tumor unit is known as BTB, which is featured by increased permeability, promoting tumor growth, and changing the delivery of anti-cancer agents. Meanwhile, dynamic angiogenesis differs from lesions and regions of the same lesion during metastatic progression (24). As a result, there is a significant heterogeneous permeability in brain metastases, leading to a non-uniform and suboptimal drug distribution and thus promotes drug resistance (28).

**Figure 2:**
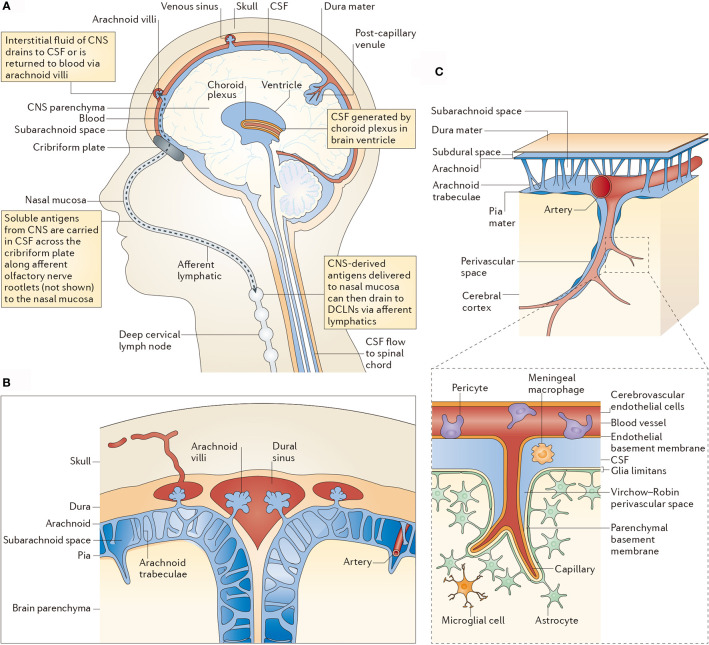
CSF-mediated drainage of interstitial fluid and CNS antigens to deep cervical lymph nodes. **(A)** A human head in midline sagittal section, showing revelant anatomical structures [namely the ventricle, choroid plexus, central nervous system (CNS) parenchyma, lymphatics and deep cervical lymph nodes (DCLNs)] in schematic form. **(B)** Arachnoid granulations in relation to the subarachnoid space and brain parenchyma. **(C)** Subpial vasculature in relation to subarachnoid space and brain parenchyma, indicating the anatomy discussed in the main text. The inset shows the cellular components of cerebral capillaries, the glia limitans and the basement membranes in relation to the perivascular space. CSF, cerebrospinal fluid.

CNS is not completely immune-privileged. In the last century, the CNS has been considered as the immune-privileged organ because of the existence of BBB and BCSFB, where immune cells in the blood circulation system are blocked. However, along with the explorations on lymphatic system in the brain and lymphatic ducts of meninges, this conception has been overthrown (29). Experimental data also showed that tumor-infiltrating T lymphocytes and other blood-borne immune cells are observed in the brain metastases (30). Besides, a connection between the blood-borne immune cells and immune components in the brain exists. The specific immune cells of the CNS are able to pass through the endolymphatic system into the cerebrospinal fluid, which further infiltrate to the olfactory bulb, olfactory nerve, cribriform plate, nasal mucosa, and finally reach the deep cervical lymph nodes. Although the BBB limits the penetration of immune cells, they can pass through the tapetum lucidum and lymphatic channels of cerebrospinal fluid (**Figure 2**) (31). Besides, macrophages and CD4-positive memory T cells are residents in the ventricle, pia mater, and perivascular space, which are important for immune monitoring of the CNS (31).

## Specific Components of Immune Microenvironment For Brain Metastases

### CNS-Derived Antigen-Presenting Cells

According to previous studies, CD11c-expressing cells are found as the residents in the juxta vascular parenchyma (32). They are not only recruited from the blood to parenchyma but also derived from an intraneural precursor *in situ* (32). Apart from the CD11c-expressing cells, perivascular and ventricular macrophages, as well as epiplexus cells of the choroid plexus and meninges constitute a main population of antigen-presenting cells (APCs) (CNS-derived APCs) (31). Although they are located outside the parenchyma, they can sample the contents of tumor cells in the parenchyma through the circulating CSF. They express MHC-II molecules, co-stimulatory molecules, and can present antigens to elicit priming and proliferation of CD4+ T cells to activate the adaptive immune response (32).

### Microglia

Microglia is another main population of APCs in CNS, playing a vital role in the immune response of CNS. It is the only one type of immune cells existed in healthy CNS parenchyma and unique in CNS. Microglial cells can respond rapidly. As the main resident immune cells in the CNS, microglia is extremely heterogeneous. Microglia can lyse tumor cells by secreting NO and sheltering the brain from the metastatic cell colonization (33). Despite their anti-tumor ability, microglial have shown their tumor-promoting effect. A previous study showed that in brain metastases initiated from lung cancer, a dense accumulation of activated microglia tightly “encapsulate” brain metastases. Microglia can respond rapidly to the metastatic lung cancer cells in the brain and lead to migration and proliferation (34). Since the morphology and molecular markers of activated microglia like MHC-II molecules, CD40 and other co-stimulatory molecules are similar to those of blood-borne macrophages, it is difficult to distinguish the two types of cells (35). Thus, they are classified into the mononuclear-macrophage system, named as microglia-macrophages. Compared with the blood-borne macrophages, microglia accounts for the minority. They express low levels of the accessory molecules required for efficient antigen presentation and present a weak antigen-presenting activity (31, 36–38). According to their functional differences, microglia can be divided into M1-like and M2-like phenotypes based on the polarization (39). As is known, M1 macrophages can either engulf tumor cells or function as APCs to provoke activate CD8+ T cells and the adaptive immune response, thus killing tumor cells. On the contrary, M2 macrophages are immunosuppressive that promote tumor growth by secreting growth factors or facilitating angiogenesis (39). A large population of microglia-macrophages in intracranial tumors are usually similar to M2 macrophages, called M2-like phenotypes. They induce tumor invasion and angiogenesis by interacting with tumor cells (28). At present, microglia in brain metastases of NSCLC have been rarely reported (34, 39). Thus, targeting microglia with M2 macrophages or inhibiting signaling pathways that activate astrocytes might provide novel ideas for immunotherapy of brain metastases of NSCLC.

### Astrocytes

Astrocytes are another glial type in CNS besides microglia, accounting for 30% of cells in CNS (40). Responding to injuries or tumors, astrocytes are activated into a reactive state that are responsible for the repair and the formation of glial scars (41). Astrocytes limit metastases without entering in the lesions in the early stage, and in turn, the inflammatory environment caused by brain metastases can activate astrocytes that further promotes tumor growth (42). Lung cancer cells employ protocadherin 7 (PCDH7) to engage astrocytes and promote the establishment of carcinoma-astrocyte gap (43). These channels allow the transfer of cGAMP from cancer cells to astrocytes, thus activating the STING pathway and promoting inflammatory cytokines released by astrocytes, including interferon-α (IFNα) and tumor necrosis factor (TNF), which is an innate immune response pathway to support tumor growth and chemoresistance (43). Besides, latest evidences have shown that activated astrocytes infiltrating to surrounding tissues are capable of triggering metastasis, in which the STAT3 signaling pathway is a key link for inhibiting intracranial metastases (44).

### Other Immune Cells

There exist a small proportion of monocytes in the CSF that comprise about 5% of cells in CSF. They derive from a minority population of CCR1+/CCR5+ monocytes in the blood circulation and are activated and retained in the CNS. For further activation, myeloid monocytes down-regulate CCR1, whereas microglia up-regulate CCR5 (40, 45).

**Figure 3:**
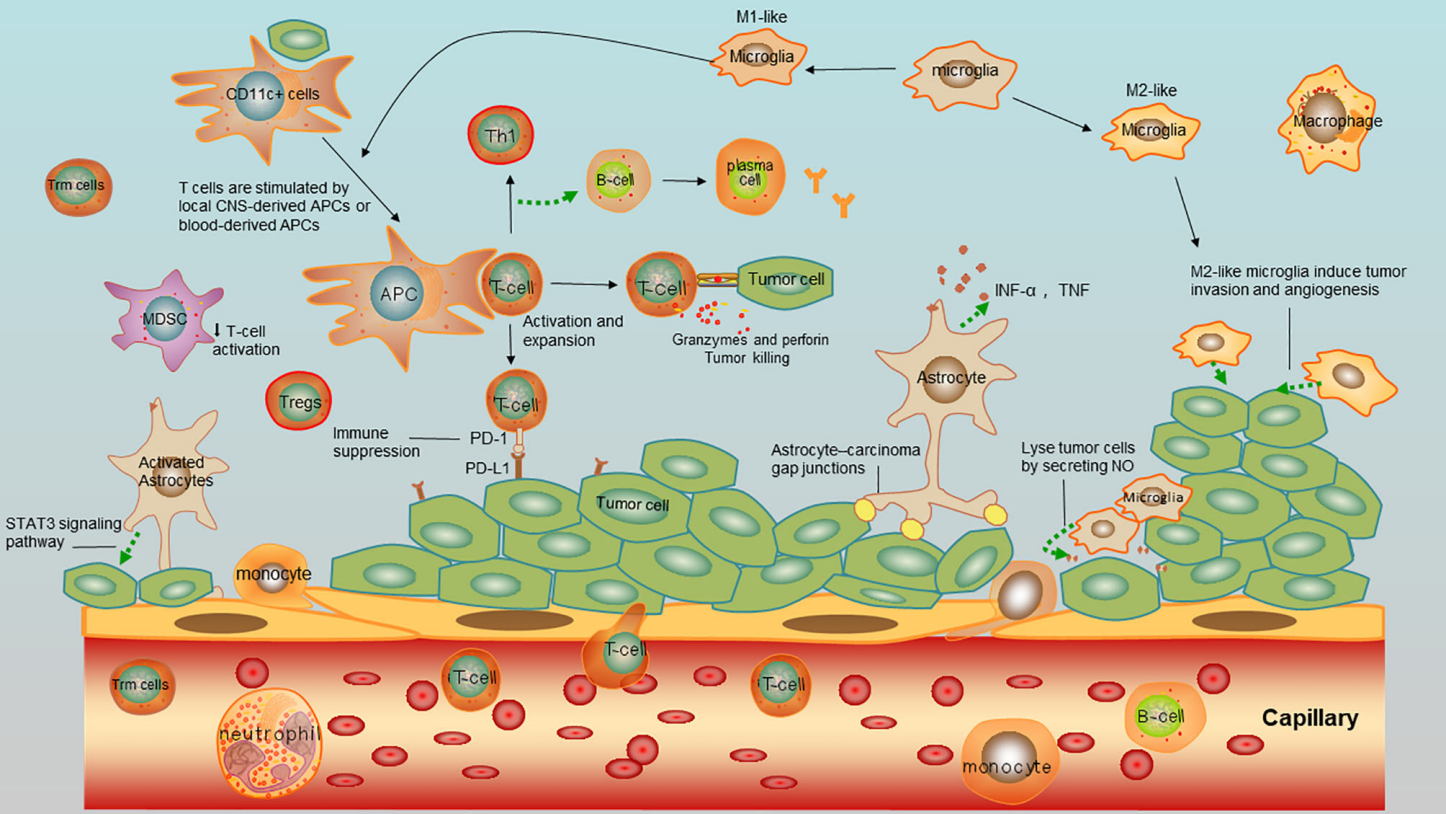
Immnue microenvirment of brain metastases of NSCLC. Tumor cells interact with tumor-infiltrating lymphocytes (TILS), antigen-presenting cells (APCs), astrocytes, microglia, myeloid derived suppressor cells (MDSCs), and macrophages.

An early study demonstrated that cerebrospinal fluid (CSF) from healthy individuals contains 1,000 to 3,000 leukocytes/ml, which predominantly consist of activated central memory T cells, suggesting that they might be involved in CNS immune surveillance (46). Moreover, it is noticed that CD8+ tissue-resident memory T (Trm) cells have been discovered in the CNS after brain viral infection (46, 47). Although the role of Trm cells in brain immune surveillance as emerged, it is not clear whether they are infiltrated during the brain metastases. To depict the components in immune microenvironment in brain metastases, the overview of immune cells and tumor cells interaction were depicted in **Figure 3**.

## Tumor-Infiltrating Lymphocytes (TILS) in Brain Metastases

As is known, TILs are the main component of tumor immune microenvironment and the key subtype of cells involved in the immune response. TILs are subtyped into two categories based on surface molecules, showing anti-cancer function and cancer-promoting effect on the immune escape, respectively (48). During the process of tumor metastasis to the brain, the damaged BBB with increased permeability allows the peripheral lymphatic system passing through the CNS. As a result, TILs can be detected around brain metastases. There are three infiltrating models of TILs, including matrix infiltration, peritumoral infiltration, and diffuse infiltration. Brain metastases of NSCLC are mainly infiltrated as the former two models (49).

## TILS for Brain Metastases Prognosis

TILs in brain metastases are positively correlated to the prognosis (12). An immunohistochemical analysis involving 61 specimens of brain metastases of lung cancer showed better overall survival in patients with higher ratios of CD3+ T cells, CD8+ T cells, and CD45+ T cells (memory T cells) than those with lower ratios of the three subtypes of T cells (30). Consistently, another study analyzing 25 pairs of primary NSCLC specimens and brain metastases of NSCLC found that the overall density of CD8+ T cells in the parenchyma of brain metastases is higher than that in primary foci, and patients with a lower number of CD8+ TILs in the matrix present a worse prognosis (50).

### Comparison Of TILs in Metastases From in Primary FOCI

TILs in brain metastases differ from those in primary foci of lung, and those in the former present a stronger immunosuppressive phenotype in the tumor microenvironment (12). In an analysis of immune gene expression profile involving 78 pairs of primary NSCLC specimens and brain metastases, a total of 161 differentially expressed genes are detected (51). Compared with primary NSCLC specimens, an attenuated antigen presentation function of dendritic cells, reduced lymphocyte extravasation and down-regulated vascular cell adhesion molecule 1 (VCAM1) are examined in brain metastases, indicating that the immunosuppressive microenvironment is more pronounced in brain metastases than that of primary foci (51). Moreover, they estimated the infiltrating level of immune cell subpopulations and lower infiltrating levels of dendritic cells, Th1 cells, and CD8+T cells are examined in brain metastases than those of primary foci. In addition, the overall ratio of infiltrating lymphocytes in brain metastases is lower than that of the primary foci, but that of macrophages is higher, especially M2 macrophages (51).

### Comparison of T Cell Receptor Characteristics in Primary Foci and Brain Metastases

It is found that both primary foci and brain metastases share the majority of tumor-associated antigens. However, the density of T cells and T-cell richness in brain metastases are significantly lower than those of primary foci (51). To further investigate T cell phenotypes, T cell clones are assessed by T cell receptor (TCR)-β sequence. In the process of TCR rearrangement, a highly variable region that recognizes antigenic peptides is formed, called complementarity determining region 3 (CDR3). TCR-β-CDR3 sequence contributes to recognize the diversity of TCR, and determines T cell clonality and abundance (52). To compare characteristics of TCR in primary foci and brain metastases, the TCR-β sequencing analysis is performed in 39 pairs of NSCLC specimens and brain metastases (51). No significant difference in the clonality among brain metastases, pulmonary primary tumors, and normal tissues is found. However, the density of T cells and clonal abundance of T cells are significantly lower in brain metastases than those of primary foci (51). Furthermore, the dominant T cell colonies are analyzed, which are shared in most brain metastases and paired primary foci, and the median ratio of shared colonies in brain metastases reaches 100%. Subsequently, the clonal proliferation of T cells in brain metastases is analyzed. Effective clonal proliferation of T cells is found in 64% of brain metastases, with a median frequency of 11.2%. In most cases, for brain metastases and primary tumors, there are tumor-associated antigens. T cell clone amplification can be detected in brain metastases, but insufficient T cell infiltrations and the diversity of TCR in metastases indicate less abundance of T cells in brain metastases (51). An analysis involving 20 cases of brain metastases of lung adenocarcinoma and primary foci consistently identified that T cell colonies and the diversity of T cells are fewer in brain metastases compared with those of primary foci (53).

### Comparison of Tumor Mutational Burden Between Primary Foci and Brain Metastases

Although tumor mutational burden (TMB) level is higher in brain metastases, the novel antigen levels do not increase and are similar to those of primary foci. Therefore, using TMB as a single immunotherapy biomarker is not reliable. Mansfield et al. further detected TMB in brain metastases of lung adenocarcinoma and primary foci (53). The average TMB of 13 cases of brain metastases of lung adenocarcinoma and primary foci is 24.9 and 12.5, respectively, indicating more non-identical mutations in intracranial lesions, which are favorable to the immune response. Later, peptides with a strong affinity to MHC are selected from the mutated sequence as new tumor antigen candidates. However, it is found that the number of new tumor antigens does not increase, which may be attributed to the limitation of current methods for predicting new antigens or short mutations generated by non-identical mutations that only a small part of them can be recognized by the immune system. Therefore, some lung adenocarcinoma patients with a relatively high TMB do not respond to immunotherapy. Moreover, it is also suggested that the use of TMB as a single immunotherapy biomarker in either primary foci or brain metastases is not reliable.

## Expression Levels of PD-1 and PD-L1 in Brain Metastases

PD-1 is mainly expressed in immune cells, including activated T cells, monocytes, and dendritic cells. After binding to PD-L1, PD-1 inactivates the cytotoxic T cells that recognize tumor cells, thus leading to the immune escape. PD-L1 is mainly expressed in tumor cells and immune cells, including T cells, B cells, macrophages, and dendritic cells (54).

PD-1 and PD-L1 are differentially expressed between brain metastases and primary foci of lung. Mansfield et al. (55) analyzed pathological samples of 73 cases of brain metastases of lung adenocarcinoma and primary foci. The positive expression of PD-L1 is detected in 39% of brain metastasis samples, while the inconsistency rate of positive expression of PD-L1 in paired cancer samples reaches 14%, and that in immune cells is 26%. It is suggested that the spatial heterogeneity of PD-L1 expression in intracranial and extracranial lesions should be taken into consideration. Notably, down-regulation of PD-L1 or loss of PD-L1 can be detected in a considerable number of intracranial lesions compared with that of primary foci. A large number of brain metastases are non-immune responded (both PD-L1 and TILs are negative).

Therapeutic strategies do not influence expression levels of PD-1/PD-L1 in brain metastases and primary foci. Preoperative radiotherapy, chemotherapy, and hormone therapy generally do not alter expression level of PD-L1 in tumor cells and immune cells of primary foci and brain metastases (56). Up-regulation of PD-1 in immune cells of brain metastases is only detected in 2/61 patients who receive preoperative radiotherapy prior to primary foci resection. Nevertheless, the small sample size limits the reliability of the conclusion that requires to be validated in large-sample studies (56).

Influences of PD-1/PD-L1 levels on therapeutic efficacy of ICI medication in brain metastases need to be further explored as well (12). Currently, a phase III clinical trial of PD-1 inhibitor in the treatment of brain metastases showed that patients with PD-L1 expression ≥ 1% in stromal/immune cells have a longer overall survival than those with PD-L1 <1% (5). Median OS is numerically higher in those with PD-L1 expression ≥ 1% in tumor cells, although no significant difference is obtainable (5). At present, there are multiple studies on immunotherapy for patients with brain metastases, and the therapeutic functions of PD-L1 are waiting to be revealed (12).

## Conclusions

As one of the most protected organs in the body, the brain is still prone to be the distant metastatic organ of NSCLC. Compared with extracranial tumors, the immune microenvironment of intracranial tumors is unique and highly specific. Specific immune cells in the immune microenvironment of intracranial tumors mainly include microglia and astrocytes, showing heterogeneous properties. Compared to the primary foci of lung, the immune microenvironment of brain metastases is overall immunosuppressed. More comprehensive and detailed studies are required to pave the way for developing new immunotherapeutic strategies by targeting their immunosuppressive properties, thus controlling brain metastases of NSCLC.

## Author Contributions

XZ, PL, and LL were major contributor in writing the manuscript. All authors contributed to the article and approved the submitted version.

## Conflict of Interest

The authors declare that the research was conducted in the absence of any commercial or financial relationships that could be construed as a potential conflict of interest.
